# Aqueous Leaf Extract of* Jatropha mollissima* (Pohl) Bail Decreases Local Effects Induced by Bothropic Venom

**DOI:** 10.1155/2016/6101742

**Published:** 2016-10-26

**Authors:** Jacyra Antunes dos Santos Gomes, Juliana Félix-Silva, Júlia Morais Fernandes, Juliano Geraldo Amaral, Norberto Peporine Lopes, Eryvaldo Sócrates Tabosa do Egito, Arnóbio Antônio da Silva-Júnior, Silvana Maria Zucolotto, Matheus de Freitas Fernandes-Pedrosa

**Affiliations:** ^1^TECBIOFAR, Department of Pharmacy, Federal University of Rio Grande do Norte (UFRN), Natal, RN, Brazil; ^2^PNBIO, Department of Pharmacy, Federal University of Rio Grande do Norte (UFRN), Natal, RN, Brazil; ^3^NPPNS, Faculty of Pharmaceutical Sciences of Ribeirão Preto, University of São Paulo, Ribeirão Preto, SP, Brazil; ^4^Federal University of Bahia, Multidisciplinary Institute in Health, Vitória da Conquista, BA, Brazil; ^5^LASID, Department of Pharmacy, Federal University of Rio Grande do Norte (UFRN), Natal, RN, Brazil

## Abstract

Snakebites are a serious worldwide public health problem. In Brazil, about 90% of accidents are attributed to snakes from the* Bothrops* genus. The specific treatment consists of antivenom serum therapy, which has some limitations such as inability to neutralize local effects, difficult access in some regions, risk of immunological reactions, and high cost. Thus, the search for alternative therapies to treat snakebites is relevant.* Jatropha mollissima* (Euphorbiaceae) is a medicinal plant popularly used in folk medicine as an antiophidic remedy. Therefore, this study aims to evaluate the effect of the aqueous leaf extract from* J. mollissima* on local effects induced by* Bothrops* venoms. High Performance Liquid Chromatography with Diode Array Detection analysis and Mass Spectrometry analysis of aqueous leaf extract confirmed the presence of the flavonoids isoschaftoside, schaftoside, isoorientin, orientin, vitexin, and isovitexin. This extract, at 50–200 mg/kg doses administered by intraperitoneal route, showed significant inhibitory potential against local effects induced by* Bothrops erythromelas* and* Bothrops jararaca* snake venoms. Local skin hemorrhage, local edema, leukocyte migration, and myotoxicity were significantly inhibited by the extract. These results demonstrate that* J. mollissima* extract possesses inhibitory potential, especially against bothropic venoms, suggesting its potential as an adjuvant in treatment of snakebites.

## 1. Introduction

Snakebites represent a serious worldwide public health and social problem because of their high frequency, morbi-mortality, and sequelae left in the victims. Moreover, accidents caused by snakes are considered a neglected disease mainly in Africa, Latin America, Asia, and Oceania [1, 2]. Data indicate that, worldwide, more than 5 million people suffer snakebites every year, resulting in 25,000 to 125,000 deaths and leaving approximately 400,000 people with permanent disabilities [1]. In Brazil, an estimated number of 25,000 snakebites occur per year [3]. Most of these accidents are caused by snake species of the* Bothrops* genus, which is responsible for about 90% of the cases in Latin America [3, 4]. The main representative species of the* Bothrops* genus in Brazil are* Bothrops jararaca* (South and Southeast),* Bothrops erythromelas* (Northeastern),* Bothrops atrox* (North), and* Bothrops moojeni* (Center-West) [3]. The* Bothrops* snakes have high complexity and variation in the protein composition of their venom. This variation is due to factors such as diet, age, seasonal variation, sexual dimorphism, and geographical origin, which occurs within the species, interfamily, intergenus, interspecies, intersubspecies, and intraspecies [5, 6]. The pathophysiological process of the snake envenoming is complex and includes the combined action of several toxins such as snake venom metalloproteinases (SVMPs), snake venom serine proteinases (SVSPs), phospholipases A_2_ (PLA_2_), hyaluronidases, bradykinin-releasing enzymes, lectins, L-amino oxidases, and pharmacological mediators [7, 8].* Bothrops* envenomation causes mainly immediate local tissue damage (including pain, edema, local hemorrhage, and myonecrosis) and systemic effects (cardiovascular alterations, coagulation, and renal alterations) [9, 10].

Currently, the intravenously antivenom serum therapy is the only specific treatment for snakebites. The antivenom, however, has some limitations such as difficult access in some regions, risk of immunological reactions (including serum sickness), high cost, and limited effectiveness in the improvement of the local tissue damage [11, 12]. Administration of the antivenom may prevent death but does not prevent local tissue damage and resultant disabilities. The low inhibition of local effects (due to the delay of receiving the serum or due to the low efficacy) is the leading cause of amputations, which can lead to serious social, economic, and health negative impacts, given that most victims live in rural areas [13]. So, the search for new complementary therapies to treat snakebites has become of utmost importance. In this scenario, the use of many medicinal plants has been an old practice in folk medicine against snakebites, especially in tropical and subtropical regions such as Africa, Asia, and South America [14, 15].

The* Jatropha *L. genus (Euphorbiaceae) belongs to the subfamily Crotonoideae and Jatropheae tribe consisting of more than 300 species and is widely distributed in the tropical and subtropical regions of Africa and the Americas [16]. The name* Jatropha* is derived from the Greek words “*jatros”* (doctor) and “*trophe”* (food), which could be correlated with the medicinal properties of plants from this genus [17]. In Africa, Asia, and Latin America, the* Jatropha *species are used not only in traditional medicine to cure various ailments, but also as ornamental plants and energy crops [17]. Several known species from genus* Jatropha *have been reported for their medicinal uses, chemical constituents, and biological activities such as* Jatropha curcas*,* Jatropha elliptica*,* Jatropha gossypifolia*, and* Jatropha mollissima* [17, 18].

*Jatropha mollissima* (Pohl) Bail (see Figure S1 in Supplementary Material available online at http://dx.doi.org/10.1155/2016/6101742) is a medicinal plant popularly known in Brazil as “pinhão-bravo” [16]. This plant is endemic in the semiarid region of Northeastern Brazil. In folk medicine, it is widely used for various purposes, especially as antiophidic [19–21] and anti-inflammatory [22] remedies, healing [20], veterinary vermifuge [19], and treatment of renal inflammation and loss of appetite [20]. Some studies have shown that this species has antioxidant [23], antimicrobial [24], and antihelmintic activities [25]. Thus, the aim of this work was to evaluate the ability of the aqueous leaf extract of* J. mollissima* to inhibit the local effects induced by* B. erythromelas* and* B. jararaca *snake venoms in mice. Our results indicate that* J. mollissima* aqueous leaf extract has significant inhibitory action against hemorrhagic, inflammatory, and myotoxic local activities of the* Bothrops* venoms studied, suggesting the potential of this species as a source of bioactive molecules against bothropic venom.

## 2. Materials and Methods

### 2.1. Chemicals and Reagents

The flavonoids apigenin (≥99%), luteolin (≥98%), luteolin-7-*O*-glucoside (≥98%), orientin (≥97%), isoorientin (≥98%), vitexin (≥95%), isovitexin (≥98%), and vitexin-2-*O*-rhamnoside (≥98%) (Sigma-Aldrich®) were purchased from Sigma-Aldrich (St. Louis, MO, USA). All other reagents and solvents used were of analytical grade. The water used was purified by reverse osmosis.

### 2.2. Plant Material

Leaves from* Jatropha mollissima* were collected in the city of “Rafael Godeiro,” 6°04′40′′S; 7°42′54′′W, RN, Brazil, in January 2014. The collection of the plant material was conducted under authorization of the Brazilian Authorization and Biodiversity Information System (SISBIO) (Process number 35017) and the Brazilian Access Authorization and Dispatch Component of the Genetic Patrimony (CGEN) (Process 010844/2013-9). The botanical identification of the material was performed by Dr. Jomar Gomes Jardim and a voucher specimen was deposited at the Herbarium of the Bioscience of the Federal University of Rio Grande do Norte, Brazil (UFRN 16879). After identification and confirmation of the plant species, the leaves were dried at room temperature, triturated with an industrial blender, and stored in hermetically sealed bottles until used for aqueous extract preparation.

### 2.3. Snake Venom

Lyophilized* B. erythromelas* and* B. jararaca* snake venoms were used in this work.* B. erythromelas* was generously supplied by the Instituto Butantan, SP, Brazil.* B. jararaca* was purchased from Sigma-Aldrich (St. Louis, MO, USA) (product number V5625). The scientific use of the material was approved by the Brazilian Access Authorization and Dispatch Component of Genetic Patrimony (CGEN) (Process 010844/2013-9). The venom was weighed and dissolved with phosphate buffer saline (PBS) and the protein content quantified by the Bradford method [26].

### 2.4. Animals

Male and female Swiss albino mice (30–35 g), 6–8 weeks of age, used in this study were maintained under standard environmental conditions with free access to water and food. On the day of the experiment, the animals were placed in the experimental room for at least one hour prior to tests, for acclimation. All animals were euthanized by sodium thiopental overdose associated with 2% lidocaine by intraperitoneal (i.p.) route, at the end of the experiments. The experimental protocols using animals were performed in agreement with the National Council for the Control of Animal Experimentation of Brazil (CONCEA) and the International Guiding Principles for Biomedical Research Involving Animals of the Council of International Organizations of Medical Sciences (CIOMS). The animal experiments were approved by the Ethics Committee on Animal Use from the UFRN (protocol number 053/2014). The total number of animals used was 230.

### 2.5. Preparation of the Aqueous Extract from the Leaves of* J. mollissima*

Dried leaves were submitted to decoction (10% w/v, plant : water) for 15 min at a temperature of around 100°C to obtain the aqueous leaf extract of* J. mollissima* (yield: 12.5% relative to dry plant). The aqueous extract obtained after vacuum filtration was freeze-dried and dissolved in PBS at adequate concentrations for the biological assays.

### 2.6. Phytochemical Analysis of the Aqueous Leaf Extract of* J. mollissima*

#### 2.6.1. TLC Profile

The extract was fractionated by liquid-liquid partition in order to obtain the dichloromethane (CH_2_Cl_2_), ethyl acetate (AcOEt),* n*-butanol (BuOH), and residual aqueous fractions, respectively. Then, the extract and fractions were analyzed by Thin Layer Chromatography (TLC) using aluminum precoated sheets with silica gel F_254_ (Merck®, Darmstadt, Germany) as adsorbent. Two different mobile phases were used: ethyl acetate : formic acid : water (8 : 1 : 1 v/v/v) and toluene : ethyl acetate : formic acid (5 : 5 : 0.5 v/v/v). The chromatograms were analyzed under 365 nm UV light and then sprayed with specific chromogenic agents according to the class of compounds investigated (Dragendorff reagent, natural reagent A, ferric chloride, and sulfuric vanillin and heating). The retention factor, behavior, and color of the spots were compared with the chromatographic profiles of the reference substances. According to the results, standard samples of the flavonoids apigenin (≥99%), luteolin (≥98%), luteolin-7-*O*-glucoside (≥98%), orientin (≥97%), isoorientin (≥98%), vitexin (≥95%), isovitexin (≥98%), and vitexin-2-*O*-rhamnoside (≥98%) (Sigma-Aldrich) were compared with the extract and fractions. When necessary to confirm the presence of any substance in extract and/or fractions, they were analyzed by co-TLC.

#### 2.6.2. HPLC-DAD Profile

The analyses were performed using an HPLC Merck-Hitach® (Hichrom model) instrument equipped with a diode array detector (DAD), quaternary pump, oven column, and autoinjector. The chromatographic analyses were performed using a Phenomenex® Luna RP-18 column (250 × 4.6 mm, 5 *μ*m particle size) and the eluents were (A) acetic acid 0.3% and (B) acetonitrile. The following gradient (v/v) was applied: 10–15% B, 0−5 min; 15-16% B, 5–50 min; 16–20% B, 50–55 min; 20% 55–75 min, 75 minutes total analysis time. Flow elution was 0.7 mL/min, and 20 *μ*L of each sample was injected. The aqueous leaf extract of* J. mollissima *was compared against standards of compounds vitexin (≥95%), isovitexin (≥98%), vitexin-2-*O*-rhamnoside (≥98%), orientin (≥97%), isoorientin (≥98%), and luteolin (≥98%) (Sigma-Aldrich). The lyophilized aqueous extract of* J. mollissima* and standards were resuspended in methanol : water, 1 : 1 (v/v). The final concentration of the extract was 2.5 mg/mL. For standards, the final concentration was 100 *μ*g/mL. Their retention time and ultraviolet spectra were obtained for chromatogram peaks at 254 and 340 nm, with the acquisition of UV spectra in the range of 200 to 400 nm. In this technique, the identification of flavonoids was based on comparison of retention time, UV spectrum of the major peaks, and coinjection of standards + extract, by the observation of the increase in the peak area. The extract was prepared in triplicate and analyzed. To confirm the presence of the standards in the aqueous extract of* J. mollissima*, coinjections of the extract with standards were performed. Acetonitrile HPLC grade purchased from Panreac® (Brazil) was used. Acetic acid was provided by Vetec® (Brazil). Water was purified with a Milli-Q system (Millipore®). After total dissolution and prior to analysis, the samples and solvents were filtered through a 0.45 *μ*m membrane (MillexTM, Merck).

#### 2.6.3. HPLC-DAD-MS/MS Analysis

To identify the chromatographic signals 1 and 2 and to confirm the signals 3, 4, 5, and 6, the aqueous leaf extract of* J. mollissima *was analyzed using a Shimadzu (Kyoto, Japan) High Performance Liquid Chromatography (HPLC) coupled to an amaZon-SL ion trap (IT) Bruker Daltonics® (Billerica, USA). The HPLC comprises a LC-20AD solvent pump unit, a CTO-20A column oven, a DGU-20A3 online degasser, a CBM-20A system controller, and a SPD-M20A (200 to 400 nm) diode array detector. Injections were performed automatically (20 *μ*L) through a 100 *μ*L loop SIL-20A HT. The separation of compounds was performed at 25°C using a Phenomenex Luna C18 column (250 mm × 4.60 mm internal diameter, 5 *μ*m particle size). The mobile phase was comprised of acetic acid 0.3% (solvent A) and acetonitrile (solvent B) and was pumped at a flow rate of 0.7 mL min^−1^. The gradient elution (v/v) program was performed as follows: 10–15% B, 0−5 min; 15-16% B, 5–50 min; 16–20% B, 50–55 min; and 20%, 55–75 min.

The mass spectrometer source parameters were set as follows: capillary voltage at 3.0 kV and end plate offset at 500 V. Nitrogen (N_2_) was used as nebulizing (60 psi) and drying gas (10 L min^−1^, 320°C). Full-scan MS and MS/MS spectra were obtained by scanning* m/z* from 50 to 1300. The electrospray ionization (ESI) source was operated in the positive and negative ionization mode. The data were acquired using amplitudes of 0.7 V (MS2) and 1.0 V (MS3). The data were processed through Bruker Compass Data Analysis 4.1 software (Bremen, Germany).

### 2.7. Inhibition of the Local Hemorrhagic Activity

The hemorrhagic activity of* B. erythromelas* and* B. jararaca* venoms was induced using the* in vivo* model of local hemorrhage, as previously described in the literature with few modifications [27]. Groups of 5 animals were treated with different doses of the extract (50–200 mg/kg, i.p.). After 30 min, the animals received a subcutaneous (s.c.) injection of 25 *µ*g of both venoms (in 100 *µ*L of PBS) in the dorsal region. 3 h later, the animals were sacrificed and had the inner surface of the skin exposed. After photo documentation of the produced hemorrhagic halos, the hemorrhagic skin was removed and weighed. The group in which animals received s.c. injection of venom and i.p. treatment of PBS was used as control (venom control). Another group that received s.c. injection and i.p. treatment of PBS was used as negative control (PBS control).

### 2.8. Inhibition of the Edematogenic Activity

The edematogenic activity of* B. erythromelas* and* B. jararaca* venoms was induced using the* in vivo* model of paw edema as previously described in the literature with few modifications [28]. Groups of 5 animals were treated with different doses of* J. mollissima* extract (50, 100, and 200 mg/kg, i.p.) or dexamethasone (2 mg/kg, i.p.). After 30 min, the animals received an intraplantar (i.pl.) injection of 1 *μ*g or 0.5 *μ*g of* B. erythromelas* or* B. jararaca *venoms, respectively, in 50 *μ*L of PBS, in the right hind paw. The individual right hind paw thickness was measured immediately before injection of the venoms (basal values) and at different time intervals (30, 60, 90, and 120 minutes) after injection of the venoms using a digital caliper (Digimess, São Paulo, SP, Brazil). A group of animals that received i.pl. injection of venoms and i.p. treatment of PBS was used as control (venom control). Another group that received i.pl. injection and i.p. treatment of PBS was used as negative control (PBS control).

### 2.9. Inhibition of the Cell Migration into Peritoneal Cavity

The ability of the* B. erythromelas* and* B. jararaca* venoms to induce migration of leukocytes into the peritoneal cavity was evaluated as described in the literature with few modifications [29]. Groups of 5 animals were treated with different doses of* J. mollissima* extract (50, 100, and 200 mg/kg i.p.) or dexamethasone (2 mg/kg i.p.). After 30 min, the animals received by i.p. route an injection of 5 *μ*g or 2.5 *μ*g of* B. erythromelas* or* B. jararaca *venoms, respectively, in 500 *μ*L of PBS. After 6 hours (*B. erythromelas* venom) or 4 hours (*B. jararaca* venom), the animals were sacrificed and the peritoneal exudates were collected through abdominal laparotomy for total leukocyte count. To facilitate the collection, all animals received an injection of 3 mL of heparinized PBS (5 IU/mL) and had the abdomen massaged to release the adhered cells. The lavage fluid was centrifuged (at 392 g for 5 min) and the cell button resuspended in 500 *μ*L of PBS. The samples were then diluted 1 : 20 in Türk solution (acetic acid and crystal violet 1%) for total leukocyte count in Neubauer's chamber. Cytospin preparations were stained with Leishman's stain for the differential cell counts. A group of animals that received i.p. injection of venoms and i.p. treatment of PBS was used as control (venom control). Another group that received i.p. injection and i.p. treatment of PBS was used as negative control (PBS control).

### 2.10. Inhibition of the Myotoxic Activity

The myotoxic activity of* B. erythromelas* and* B. jararaca* venoms was induced using the serum creatine kinase (CK) level, as previously described in the literature with few modifications [28]. Groups of 5 animals were treated with different doses of* J. mollissima* extract (50–200 mg/kg, i.p.) or dexamethasone (2 mg/kg, i.p.). After 30 min, all the animals received an intramuscular (i.m.) injection of 25 *µ*g of both venoms (in 50 *µ*L of PBS) in the right thigh. 3 h later, the animals were anesthetized with sodium thiopental and the blood was collected. The blood samples were incubated for 10 min at 37°C and, then, centrifuged at 10,000 g for 10 min to obtain the serum. The serum CK activity was determined using a commercial kit according to the manufacturer's protocol adapted for reading in the microplate reader (Epoch-Biotek, Winooski, VT, USA). A group in which animals received i.m. injection of venom and i.p. treatment of PBS was used as control (venom control). Another group that received s.c. injection and i.p. treatment of PBS was used as negative control (PBS control).

### 2.11. Statistical Analysis

All results were presented as mean ± standard error of mean (SEM). One-way ANOVA with Tukey's posttest and regression analysis were performed using GraphPad Prism version 5.00 (San Diego, CA, USA).* p* values less than 0.05 were considered significant.

## 3. Results

### 3.1. Phytochemical Analysis of the Aqueous Extract of* J. mollissima*

#### 3.1.1. Thin Layer Chromatography (TLC) Profile

For phytochemical analysis by TLC, the aqueous extract was fractionated by liquid-liquid partition to obtain fractions with different polarities, thus facilitating the chromatographic analysis of the compounds. Moreover, the TLC analysis was performed with different developers in order to identify the classes of compounds presented in the aqueous extract of* J. mollissima*. The chromatograms show spots suggestive of the presence of phenolic compounds, flavonoids, and saponins (Figure S2). By co-TLC analysis, it was possible to observe the presence of the flavonoids apigenin (*Rf* 0.64, fluorescent green color), luteolin (*Rf* 0.64, fluorescent yellow color), orientin (*Rf* 0.64, fluorescent yellow color), isoorientin (*Rf* 0.64, fluorescent yellow color), and vitexin (*Rf* 0.64, fluorescent green color) in the extract (Figure S3). For confirmation of presence of such substances, analyses were performed by HPLC.

#### 3.1.2. High Performance Liquid Chromatography (HPLC-DAD) Profile

**Figure 1:**
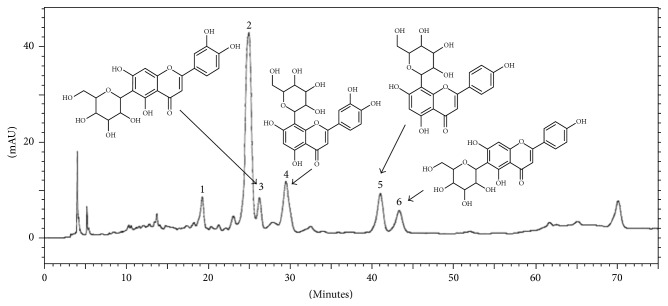
High Performance Liquid Chromatography of the aqueous extract of* J. mollissima.* SP: Phenomenex Luna C18 column (250 × 4.6 mm, 5 *μ*m); MP: ACN gradient: acetic acid 0.3%; flow rate: 0.7 mL/min; detection: 340 nm. Four compounds were identified as isoorientin (peak 3), orientin (peak 4), vitexin (peak 5), and isovitexin (peak 6).

The chromatographic fingerprint obtained by HPLC-DAD of aqueous extract of* J. mollissima* is depicted (Figure 1). It is possible to observe that* J. mollissima* exhibits at least six major peaks (1–6). Among them, most have UV spectra similar to glycosylated flavonoid derivatives from apigenin (267 nm II band and 336 nm I band) and luteolin (253 and 267 nm II band and 349 nm I band) [30]. Glycosylated derivatives from these two flavonoids, orientin and isoorientin (derived from luteolin) and vitexin and isovitexin (derived from apigenin), have similar absorption II bands for these aglycones, differing mainly by the maximum absorption of the I band. Thereafter, by the analysis of standards and coinjection of extract + standard, it was possible to observe the increase in peak area of each standard analyzed. The peaks 3, 4, 5, and 6 had their identity confirmed as, respectively, isoorientin (*tR* 24.95 min, UV 269 and 349 nm), orientin (*tR *27.96 min, UV 256 and 348 nm), vitexin (*tR *39.8 min, UV 267 and 336 nm), and isovitexin (*tR *41.35 min, UV 269 and 337 nm). Peaks 1 (*tR *24.9 min, UV 271 and 335 nm) and 2 (*tR *26 min, UV 269 and 347 nm) could not be identified by coinjection.

#### 3.1.3. HPLC-DAD-MS/MS Analysis

**Figure 2:**
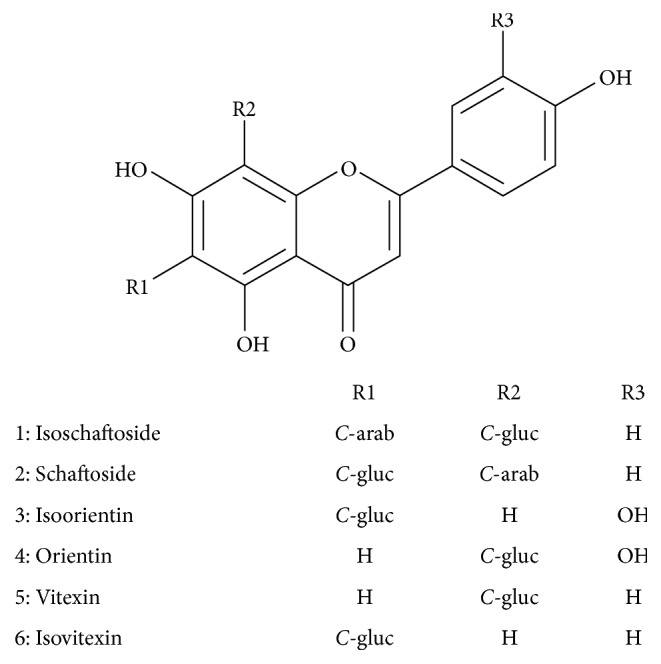
Flavonoids from* J. mollissima.*

Mass Spectrometry analysis of signals 1 and 2 showed the same ion formation at* m/z* 563.2 [M−H]^−^ in negative mode and* m/z* 565.2 [M+H]^+^ in positive mode. The MS/MS spectra exhibited fragmentation patterns of the isomers apigenin-di-*C*-glycoside by the presence of ions [M–90]^+/−^ for* C*-pentosides and [M−120]^+/−^ for* C*-hexosides and fragment ions [Agl+83/113]^−^, which for di-*C*-glycosides represent the aglycone, and the residues of the sugars that remained linked to it identified the type of aglycone, that is, [311/341]^−^ and [353/383]^−^ for apigenin and [327/357]^−^ and [369/399]^−^ for luteolin, mono-*C*, and di-*C*-glycosides, respectively [31]. Applying the systematic analyses carried out by Ferreres and coworkers [31, 32], it was possible to identify the compounds as schaftoside and isoschaftoside flavonoid, respectively. According to such report, preferential fragmentation is of the sugar moiety at the 6-*C *rather than the 8-*C* position. Observed in the present study was the formation of ions* m/z* 473 [(M−H)−90]^−^ as the base peak of chromatographic signal 1 and* m/z* 473 [(M−H)−120]^−^ as the base peak of chromatographic signal 2 (Table 1). Considering the preferential fragmentation at the* C*-6 position, chromatographic signal 1 corresponded to the isoschaftoside flavonoid and chromatographic signal 2 corresponded to the schaftoside flavonoid. Furthermore, peaks 3, 4, 5, and 6 were confirmed as isoorientin, orientin, vitexin, and isovitexin as suggested by the coinjection performed with these standards (see Section 3.1.2). All the signals present in Table 1 are in full agreement with the previous published data.  Figure 2 shows flavonoids identified by HPLC-DAD-MS/MS for the species* J. mollissima*.

### 3.2. Inhibition of the Local Hemorrhagic Activity

**Figure 3:**
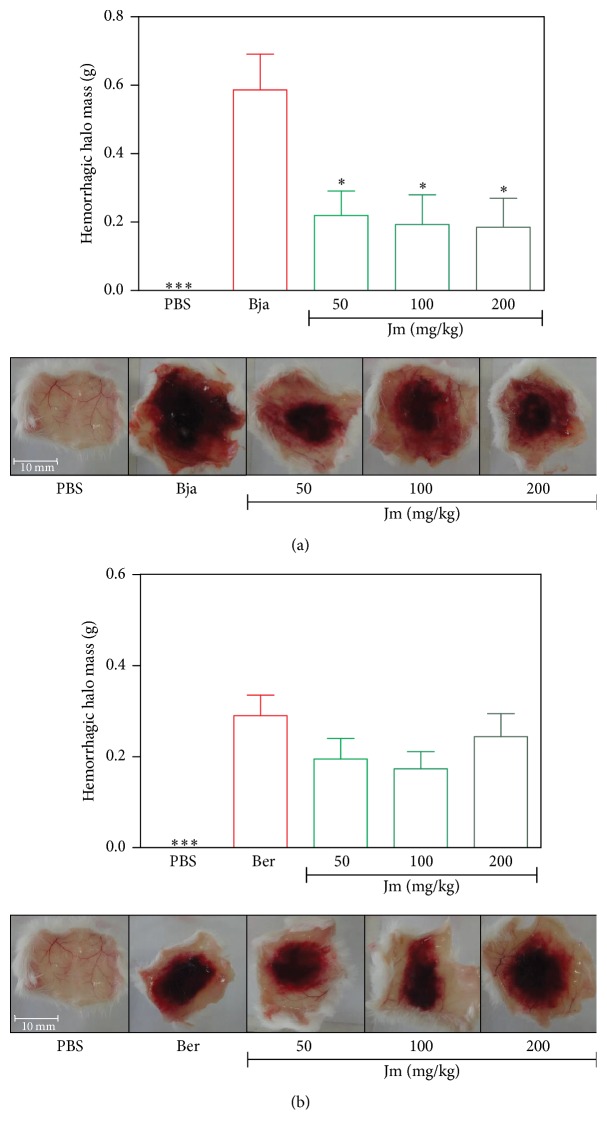
Inhibition of the hemorrhagic activity of* B. jararaca* and* B. erythromelas *venoms by aqueous extract of* J. mollissima *(Jm). (a)* B. jararaca* (Bja) and (b)* B. erythromelas* (Ber) venoms were injected s.c. in the ventral region of the animals treated with different i.p. doses of Jm. 3 h later, the inner surface skin was exposed, photo documented, and the halos weighed. Values were expressed as mean ± standard error of mean with *n* = 5. ^*∗*^ *p* < 0.05 and ^*∗∗∗*^ *p* < 0.001 when compared to the venom control group (Tukey's test, one-way ANOVA).

The subcutaneous injection of* B. jararaca* and* B. erythromelas* venoms induced severe hemorrhagic damage surrounding the ventral region after 3 h, compared to the subcutaneous injection of PBS (*p* < 0.05) (Figures 3(a) and 3(b)). The* B. jararaca* venom proved to be more potent in causing hemorrhage in relation to the* B. erythromelas* venom, using equal doses per animal. All doses of the* J. mollissima* extract (50–200 mg/kg) inhibited the local hemorrhagic activity caused by the* B. jararaca* venom (*p* < 0.05), as shown in Figure 3(a). The maximum inhibition by extract was 44% at a dose of 200 mg/kg for 3 hours. This result can be seen with the decrease in hemorrhagic halo weight and with the visual decreased halo diameter, in each dose tested. On the other hand, for* B. erythromelas*, it was observed that the hemorrhage caused by this venom was reduced by the extract, particularly at doses of 50 and 100 mg/kg, but this decrease was not statistically significant (*p* > 0.05) (Figure 3(b)).

### 3.3. Inhibition of the Edematogenic Activity

**Figure 4:**
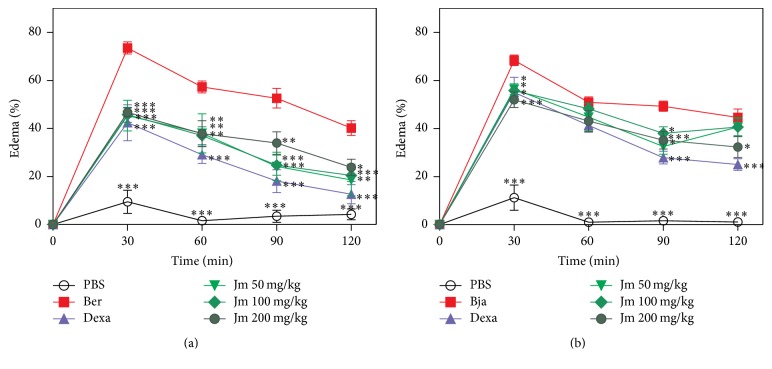
Inhibition of the edematogenic activity of* B. erythromelas* (a) and* B. jararaca *(b) by aqueous leaf extract of* J. mollissima* and dexamethasone. Values were expressed as mean ± standard error of mean with *n* = 5. ^*∗*^ *p* < 0.05, ^*∗∗*^ *p* < 0.01, and ^*∗∗∗*^ *p* < 0.001 when compared to the venom control group (Bonferroni's test, two-way ANOVA).

*B. erythromelas* and* B. jararaca* venoms showed a marked edematogenic effect for 120 min after intraplantar injection, compared to PBS (*p* < 0.05) (Figure 4). The* B. jararaca* venom proved to be more potent in causing edema in relation to the* B. erythromelas* venom, since a lower dose of* B. jararaca* (0.5 *μ*g/paw) produced an effect similar to that of the* B. erythromelas* venom (1.0 *μ*g/paw). A lower dose of the* B. jararaca* venom was used since this venom is very hemorrhagic and we intended to evaluate edema dissociated from hemorrhage. The treatment with dexamethasone significantly reduced the edema induced by both venoms. It could be observed that the* J. mollissima* extract (50–200 mg/kg), administered half an hour before the injection of the* B. erythromelas* (Figure 4(a)) and the* B. jararaca* (Figure 4(b)) venoms, inhibited the edematogenic activity after 120 min of the venom injection (*p* < 0.05). For* B. erythromelas*, the maximum inhibition by extract was 46.41% at a dose of 50 mg/kg for 120 min while for* B. jararaca* the maximum inhibition by extract was 25.19% at a dose of 200 mg/kg for 120 min. Maximum inhibition for dexamethasone was 57.58% for* B. erythromelas* and 30.65% for* B. jararaca.*

### 3.4. Total Number of Leukocytes in the Peritoneal Cavity

**Figure 5:**
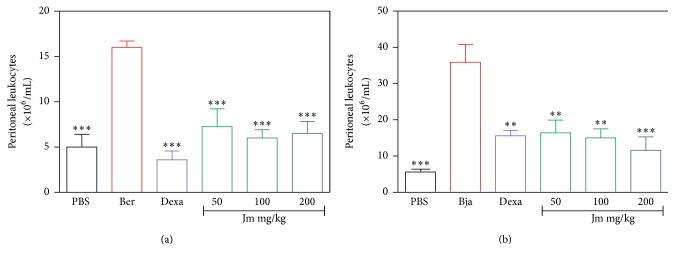
Evaluation of the inhibition by the* J. mollissima* extract of the cell migration induced by (a)* B. erythromelas* and (b)* B. jararaca* venoms. Evaluation of the total leukocyte influx into peritoneal cavity after venom injection. Values were expressed as mean ± standard error of mean with *n* = 5. ^*∗∗*^ *p* < 0.01 and ^*∗∗∗*^ *p* < 0.001 when compared to the venom control group (ANOVA).

Leukocyte infiltration was evaluated in the peritoneal cavity at the time of 4 hours after the intraperitoneal injection of the venoms. The group with the injected venoms showed an increase in the number of white cells compared to the control PBS group count (*p* < 0.05). The* B. jararaca* venom proved to be more potent in causing leukocyte infiltration in relation to the* B. erythromelas* venom. Figures 5(a) and 5(b) show the total number of leukocytes in the peritoneal cavity of the animals treated with the* J. mollissima* extract half an hour before the intraperitoneal injection of the* B. erythromelas* venom and the* B. jararaca* venom, respectively. In both cases, all tested doses of the* J. mollissima* extract (50–200 mg/kg) and dexamethasone were able to reduce the leukocyte influx compared to the control group (which received only venom) (*p* < 0.05).

**Figure 6:**
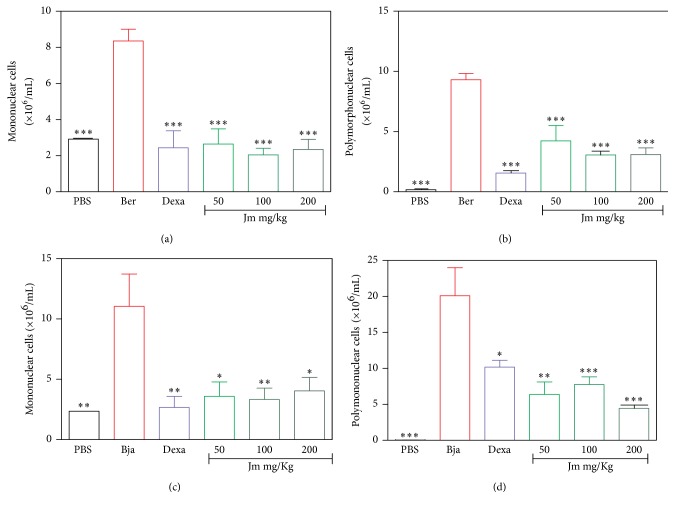
Evaluation of the inhibition by the* J. mollissima* extract of the cell migration induced by* B. erythromelas *(a, b) and* B. jararaca *venoms (c, d). (a, c) Mononuclear cell count. (b, d) Polymorphonuclear cell count. Evaluation of the leukocyte influx after treatment with the extract of* J. mollissima* (Jm) (50, 100, and 200 mg/kg, i.p.) and dexamethasone (Dexa) (2 mg/kg, i.p.). Values were expressed as mean ± standard error of mean with *n* = 5. ^*∗*^ *p* < 0.05, ^*∗∗*^ *p* < 0.01, and ^*∗∗∗*^ *p* < 0.001 when compared with venom control group (ANOVA).

The differential count of the leukocyte infiltration was evaluated.* J. mollissima* extract (50–200 mg/kg) or dexamethasone significantly reduced the influx of mononuclear (Figures 6(a) and 6(c)) and polymorphonuclear (Figures 6(b) and 6(d)) cells induced by both venoms (*p* < 0.05). All the results show that the animals treated with the* J. mollissima* extract or dexamethasone half an hour before the intraperitoneal injection of the venoms significantly inhibited the migration of these cells into the peritoneal cavity compared to the control group (which received only the venoms) (*p* < 0.05). For* B. erythromelas*, the maximum inhibition by extract was 80% at a dose of 100 mg/kg while for* B. jararaca* the maximum inhibition by extract was 80.18% at a dose of 200 mg/kg. Maximum inhibition for dexamethasone was 87.27% for* B. erythromelas* and 66.97% for* B. jararaca.*

### 3.5. Inhibition of the Myotoxic Activity

**Figure 7:**
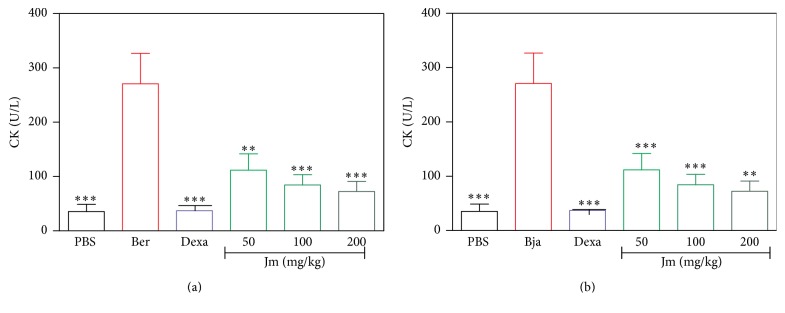
Inhibition of the myotoxic activity of* B. erythromelas* (a) and* B. jararaca *(b) by aqueous leaf extract of* J. mollissima*.* B. erythromelas *(Ber) and* B. jararaca* venoms (Bja) were injected i.m. in the left thigh of animals treated with different i.p. doses of the* J. mollissima* (Jm) extract. 3 h later, the blood was collected for creatine kinase (CK) determination. Values were expressed as mean ± standard error of mean with *n* = 5. ^*∗∗*^ *p* < 0.01 and ^*∗∗∗*^ *p* < 0.001 when compared with venom control group (ANOVA).

The intramuscular injection of both venoms induced a significant increase in the CK in serum after 3 h, compared to PBS (*p* < 0.05) (Figures 7(a) and 7(b)). Treatment with dexamethasone reduced serum CK induced by these venoms (*p* < 0.05). In the same way, treatment with extract (50–200 mg/kg) showed significant reduction in the serum CK levels induced by the* B. erythromelas* (Figure 7(a)) and the* B. jararaca* (Figure 7(b)) venoms (*p* < 0.05). For* B. erythromelas*, the maximum inhibition by extract was 81.70% at a dose of 200 mg/kg while for* B. jararaca* the maximum inhibition by extract was 72.73% at a dose of 100 mg/kg. Maximum inhibition for dexamethasone was 92.66% for* B. erythromelas* and 96.10% for* B. jararaca*.

Table 2 summarizes the maximum percentage of inhibition of the* J. mollissima* extract in the hemorrhagic, edematogenic, inflammatory, and myotoxic activities induced by* B. erythromelas* and* B. jararaca* venoms.

## 4. Discussion

Currently, the only available specific treatment for snakebites is the antivenom serum therapy, which has some limitations, such as reduced effectiveness against local effects, risk of immunological reactions, high cost, and difficult access in some regions [11, 12]. Even though the antivenom causes the inhibition of systemic effects, the neutralization of the local tissue damage is much more difficult [4, 28]. Given these limitations, it is important to find alternative treatments and/or complementary therapies. In this context, the use of medicinal plants could be highlighted, since many of them could be able to neutralize a broad spectrum of toxins (including the local tissue damage) [28, 33]. Indeed, several medicinal plants are rich sources of natural inhibitors and have pharmacologically active components. They are also able to be stable at room temperature, having easy access and low cost [34].

In this work, a phytochemical study of* J. mollissima* was conducted for better comprehension of the chemical compounds presented in the aqueous extract of leaves of this species. The TLC analysis identified the presence of flavonoids that could be suggested as major compounds, judging by the number, size, and intensity of spots when revealed with Reagent A Natural (specific spray reagent for this class of compounds). By the HPLC-DAD and HPLC-DAD-MS/MS analysis, the presence of six flavonoids (isoschaftoside, schaftoside, isoorientin, orientin, vitexin, and isovitexin) was confirmed. These flavonoids have been described in the literature for this species and some others of the* Jatropha* genus [16, 35–37]. These results are interesting, since several studies show that flavonoids have significant inhibitory activities against some snake venom enzymatic toxins such as PLA_2_ and hyaluronidases [38, 39]. These compounds could inhibit snake venom toxins directly, acting as enzyme inhibitors and chemical inactivators, or indirectly as immunomodulators, interacting with biological targets [38]. Additionally, these phenolic compounds are capable of chelating metal ions, which are essential for the activity of toxins such as SVMPs and PLA_2_ [34, 38]. Among the flavonoids detected in* J. mollissima *aqueous leaf extract, the presence of glycosylated derivatives from luteolin could be highlighted, as these compounds have been shown to possess antiophidic properties [40, 41]. Therefore, a plausible hypothesis is that the major compounds of the* J. mollissima* extract could be responsible for the inhibitory properties experimentally observed in this work. To confirm such assumption, the isolation of these compounds is currently underway in our group.

Local hemorrhage is one of the main symptoms of bothropic envenomation [42]. The hemorrhagic SVMPs (hemorrhagins) are the main compounds responsible for this effect, causing proteolysis of basal lamina components of the microvasculature leading to a rupture of the blood vessels with the appearance of fissures, which will result in leakage of blood to the exterior [43]. Additionally, SVMPs are responsible for hydrolyzing proteins in the cell membrane, such as integrins, cadherins, collagen type IV, laminin, and fibronectin [42]. Indeed, SVMPs are the key enzymes that contribute to the toxicity of* Bothrops* genus, as it has been estimated that they comprise at least 30% of the total protein content [7]. SVMPs are zinc-dependent enzymes with molecular masses that range from 20 to 100 kDa and play different functions in snake envenoming such as proteolytic degradation, alterations in blood coagulation, proinflammatory activity, inhibition of platelet aggregation, and myotoxic and myonecrotic activity. The SVMPs are divided into three classes and several subclasses: PI SVMPs (no hemorrhagic activity), PII SVMPs (disintegrin domain, with proteolytic activity), and PIII SVMPs (disintegrin domain and cysteine-rich domain, being the most hemorrhagic) [7, 44, 45]. To date, several SVMPs, from PI, PII, and PIII classes, have been purified and/or characterized in* B. jararaca* venom [7, 44], while in* B. erythromelas* venom, only a very few SVMPs have already been characterized, being PI and PIII SVMPs [46]. This study revealed that the* J. mollissima* extract was able to inhibit the hemorrhage caused by the* B. jararaca* venom. In fact, through the external appearance of the hemorrhagic halos, a decrease in the hemorrhage according to the increase of the extract dose was observed (Figure 3(a)). So, this result could indicate an inhibitory action upon the SVMP action. On the other hand, the* J. mollissima* extract was also able to inhibit the hemorrhage caused by the* B. erythromelas* venoms, but this decrease was not significant (Figure 3(b)). This may be due to the different composition of SVMPs in both venoms and the possible selectivity of the extract against SVMPs from the* B. jararaca* venom

Edema is one of the first effects caused by the bothropic envenoming. Various toxins may be responsible for edematogenic activity produced by bothropic venoms including Asp 49 or Lys 49 PLA_2_ and hemorrhagic or nonhemorrhagic SVMPs [47, 48]. This activity is the result of combined action of various toxins found in* Bothrops* venoms, acting rapidly in the connective and muscle tissue, inducing the release of various endogenous inflammatory mediators. This is the reason for the decrease in the efficacy of conventional antivenom serum therapy against these local inflammatory reactions [4, 49]. In fact, this therapy is able to neutralize the toxins but cannot neutralize the effects produced by the endogenous inflammatory mediators [49, 50]. Moreover, the injection of the* B. jararaca* venom in mice paws induces edema, which is mainly mediated by the metabolites of the arachidonic acid and the involvement, in a low level, of histamine, serotonin, and platelet-activating factor [51, 52]. SVMPs also have an important role in the inflammatory response by degrading the extracellular matrix, an effect that can affect wound healing and tissue regeneration [48]. The results obtained in this study revealed that* J. mollissima* extract efficiently inhibited the edematogenic activity produced by* B. erythromelas* (Figure 4(a)) and* B. jararaca* (Figure 4(b)) venoms after 120 minutes. This result was similar to that produced by dexamethasone, which is a steroidal anti-inflammatory drug widely used in medical practices. Dexamethasone inhibits the PLA_2_ and, consequently, there is a decrease in the production of the products derived from the arachidonic acid, which is generated by the cyclooxygenase and the lipoxygenase route [49]. Previous studies show that dexamethasone decreased the acute inflammatory response induced by the* Bothrops moojeni* in mice because of its ability to decrease the formation of eicosanoids in the presence of the venom [49, 50]. So, in this context, two explanations could be addressed for the inhibitory effect presented by the* J. mollissima* extract: it could be directly inhibiting the toxins involved in the inflammatory effect produced by the venoms and/or it could be acting as a potent anti-inflammatory agent.

After the formation of the edema, the next local reaction is the recruitment of leukocytes, which selectively migrate to the site of the inflammation [53]. In the acute inflammatory response, there is a predominant accumulation of neutrophils. These cells represent the first line of defense in the body and have a phagocytic capacity for the removal of the aggressor agent. In the later stages of the inflammatory response, mononuclear cells are observed [54]. Previous studies have shown that the metalloproteinases, present in the* B. asper* venom, when injected into the peritoneal cavity of mice, induced an increase in the IL-1 levels, followed by an increased expression of adhesion molecules. These metalloproteases were also responsible for the activation of the complement system, resulting in an increase in the cell migration [55, 56].* Bothrops* venom also possesses PLA_2_ toxins, which are important for the induction of the leukocyte migration, most likely by inducing more potently the release of proinflammatory mediators [57, 58]. In this study, an increase in the leukocytes was observed in the peritoneal cavity after 6 hours and 4 hours induced by* B. erythromelas* and* B. jararaca* venoms, respectively. Similar to the dexamethasone,* J. mollissima* extract efficiently inhibited the number of total leukocytes (Figures 5(a) and 5(b)) and the number of mononuclear and polymorphonuclear cells that migrated into the peritoneal cavity induced by the* B. erythromelas* and* B. jararaca* venoms at all tested doses (Figures 6(a), 6(b), 6(c), and 6(d)). Therefore, it can be suggested that the* J. mollissima* extract is inhibiting the Asp 49 and/or the Lys 49 PLA_2_ or that the* J. mollissima* extract has a potent inhibitory effect against the endogenous chemical mediators released by the action of the toxins. Another possibility may be an inhibitory action upon SVMPs, since the* J. mollissima* extract also presented an antihemorrhagic effect (Figure 3).

Damage to the muscle tissue (myonecrosis) is a serious local effect of the bothropic envenomation, since it can lead to permanent loss of tissue, disability, and even amputation [59, 60]. The myotoxicity may be due to a direct action of the Asp 49 or the Lys 49 PLA_2_, which directly injures skeletal muscle cells, affecting the integrity of their plasmatic membrane. The PLA_2_ damages the sarcolemma, resulting in a loss of calcium permeability and, consequently, causing rupture of this membrane, leading to a rapid release of the cytosolic markers such as lactate dehydrogenase (LDH) and creatine kinase (CK) [55, 61]. SVMPs can lead to myotoxicity by an indirect action, due to the ischemia caused by the vascular disorders resulting from hemorrhagic action, which can lead to muscle necrosis and the consequent release of CK [4, 61]. The inflammatory reaction induced by the snake venoms contributes to further development of muscle damage [62, 63]. Patrão-Neto et al. (2013) [49] demonstrated that dexamethasone decreased the late myotoxicity triggered by bothropic venom, since this compound has significant anti-inflammatory properties. In this present work,* J. mollissima* extract was able to decrease CK levels in animals injected with* B. erythromelas* and* B. jararaca* venoms, as shown in Figures 7(a) and 7(b), respectively. This result was similar to the anti-inflammatory drug dexamethasone, which possibly inhibits the inflammatory effects produced by* Bothrops* venoms. This result suggests that the extract possesses antimyotoxic action, possibly by inhibiting inflammation induced by the venom, since inflammation is an important finding in the local muscle damage. Furthermore, a possible inhibition of the direct action of PLA_2_ could be suggested in addition to this anti-inflammatory effect. Additionally, the action of the extract on the indirect myotoxic action of SVMPs could be supported since the extract also presented an antihemorrhagic effect.

## 5. Conclusions

Overall, these results demonstrate the potential of the* J. mollissima* extract in the treatment of the local effects produced by bothropic venoms. It could be concluded that probably the aqueous extract from the leaves of* J. mollissima* has substances that can inhibit or inactivate the toxins presented in the* B. erythromelas* and the* B. jararaca* venoms, as well as acting indirectly upon endogenous mediators. Therefore, the set of results provides scientific evidence of the potentiality of the* J. mollissima* extract. Other experimental models should be tested by our research group in order to suggest the usefulness of this plant as a future potential adjuvant in the treatment of local effects due to snakebites, along with antivenom therapy.

## Supplementary Material

Figure S1: Jatropha mollissima (Pohl) Bail is a medicinal plant popularly known in Brazil as “pinhão-bravo”. J. mollissima were collected in the city of “Rafael Godeiro,” 6°04'40"S; 7°42'54''W, located in Rio Grande do Norte State, RN, Brazil. Photography by Jacyra A.S. Gomes.
Figure S2: The aqueous extract and fractions were analyzed by Thin Layer Chromatography (TLC) using aluminum pre-coated sheets with silica gel F254 (Merck, Darmstadt, Germany) as adsorbent. Two different mobile phases were used: (1) ethyl acetate: formic acid: water (8:1:1 v/v/v) and (2) toluene: ethyl acetate: formic acid (5:5:0.5 v/v/v). The chromatograms were analyzed under 365 nm UV light and then sprayed with specific chromogenic agents according to the class of compounds investigated (sulfuric vanillin + heating, natural reagent A, ferric chloride, and Dragendorff reagent). Photography by Jacyra A.S. Gomes.
Figure S3: co-TLC analysis, it was possible to observe the presence of the flavonoids apigenin (Rf 0,64, fluorescent green color), luteolin (Rf 0,64, fluorescent yellow color), orientin (Rf 0,64, fluorescent yellow color), isoorientin (Rf 0,64, fluorescent yellow color), and vitexin (Rf 0,64, fluorescent green color) in the extract. Photography by Jacyra A.S. Gomes.

## Figures and Tables

**Table 1:** Identification de *C*-glycosyl and di-*C*-glycosyl flavones and relative abundances of ions obtained from the respective ions [M–H]^−^ by MS/MS.

Peak	Identification	[M−H]^−^	MS/MS [M−H]^−^					
			−18	−60	−90	−120	A + 113	A + 83
1	Isoschaftoside	563	545^(12)^	503^(39)^	473^(100)^	443^(88)^	383^(51)^	353^(68)^
2	Schaftoside	563	545^(19)^	503^(40)^	473^(84)^	443^(100)^	383^(40)^	353^(54)^
3	Isoorientin	447	429^(24)^	—	357^(87)^	327^(100)^	—	—
4	Orientin	447	—	—	357^(47)^	327^(100)^	—	—
5	Vitexin	431	—	—	341^(6)^	311^(100)^	—	—
6	Isovitexin	431	413^(6)^	—	341^(31)^	311^(100)^	—	—

**Table 2:** Maximum percentage of inhibition of *J. mollissima* against local effects produced by *Bothrops erythromelas* and *B. jararaca *venoms. Percentage of inhibition was calculated as [1 – (% activity of test mean – % activity of PBS control mean)/(% activity of Ber or Bja control mean – % activity of PBS control mean)] × 100.

	Activity	Dose	Inhibition (%)
*Bothrops erythromelas*	Edematogenic^*∗*^	50 mg/kg	46.41
	Inflammatory	100 mg/kg	81.81
	Myotoxic	200 mg/kg	81.70

*Bothrops jararaca*	Hemorrhagic	200 mg/kg	44.00
	Edematogenic^*∗*^	200 mg/kg	25.19
	Inflammatory	200 mg/kg	80.18
	Myotoxic	100 mg/kg	72.73

^*∗*^The edematogenic activity was calculated using the area under the curve.
