# Supplementary material for: Aqueous Leaf Extract of Jatropha mollissima (Pohl) Bail Decreases Local Effects Induced by Bothropic Venom
Source: Biomed Res Int. 2016 Oct 26;2016:6101742. doi: 10.1155/2016/6101742 (PMC5101363; doi:10.1155/2016/6101742)
Supplement: Supplementary file 1 — Figure S1: Jatropha mollissima (Pohl) Bail is a medicinal plant popularly known in Brazil as “pinhão-bravo”. J. mollissima were collected in the city of “Rafael Godeiro,” 6°04'40"S; 7°42'54''W, located in Rio Grande do Norte State, RN, Brazil. Photography by Jacyra A.S. Gomes. Figure S2: The aqueous extract and fractions were analyzed by Thin Layer Chromatography (TLC) using aluminum pre-coated sheets with silica gel F254 (Merck, Darmstadt, Germany) as adsorbent. Two different mobile phases were used: (1) ethyl acetate: formic acid: water (8:1:1 v/v/v) and (2) toluene: ethyl acetate: formic acid (5:5:0.5 v/v/v). The chromatograms were analyzed under 365 nm UV light and then sprayed with specific chromogenic agents according to the class of compounds investigated (sulfuric vanillin + heating, natural reagent A, ferric chloride, and Dragendorff reagent). Photography by Jacyra A.S. Gomes. Figure S3: co-TLC analysis, it was possible to observe the presence of the flavonoids apigenin (Rf 0,64, fluorescent green color), luteolin (Rf 0,64, fluorescent yellow color), orientin (Rf 0,64, fluorescent yellow color), isoorientin (Rf 0,64, fluorescent yellow color), and vitexin (Rf 0,64, fluorescent green color) in the extract. Photography by Jacyra A.S. Gomes. [file 6101742.f1.docx]

Supplementary Materials


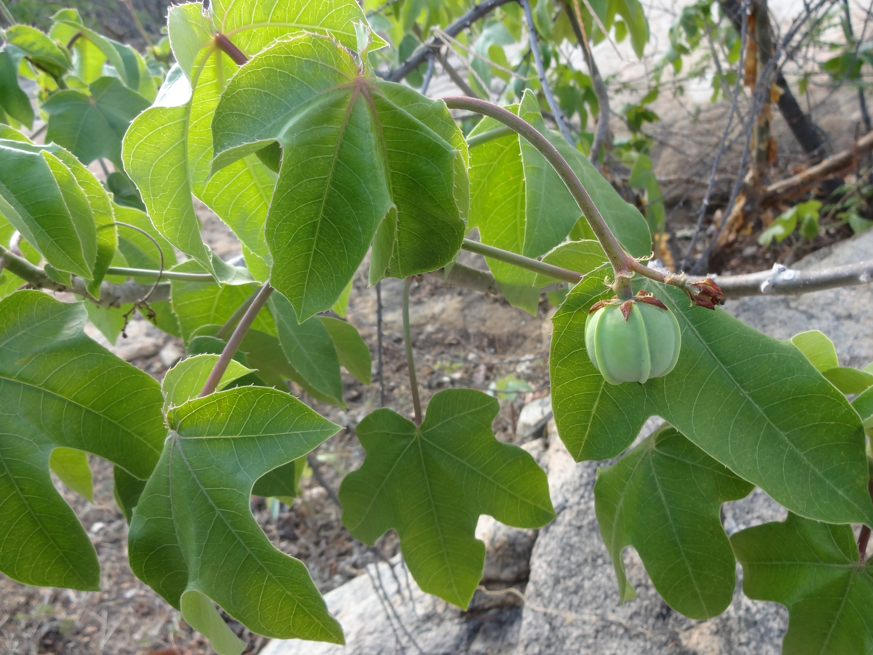


**Figure S1:** *Jatropha mollissima* (Pohl) Bail. Photography by Jacyra A.S. Gomes.


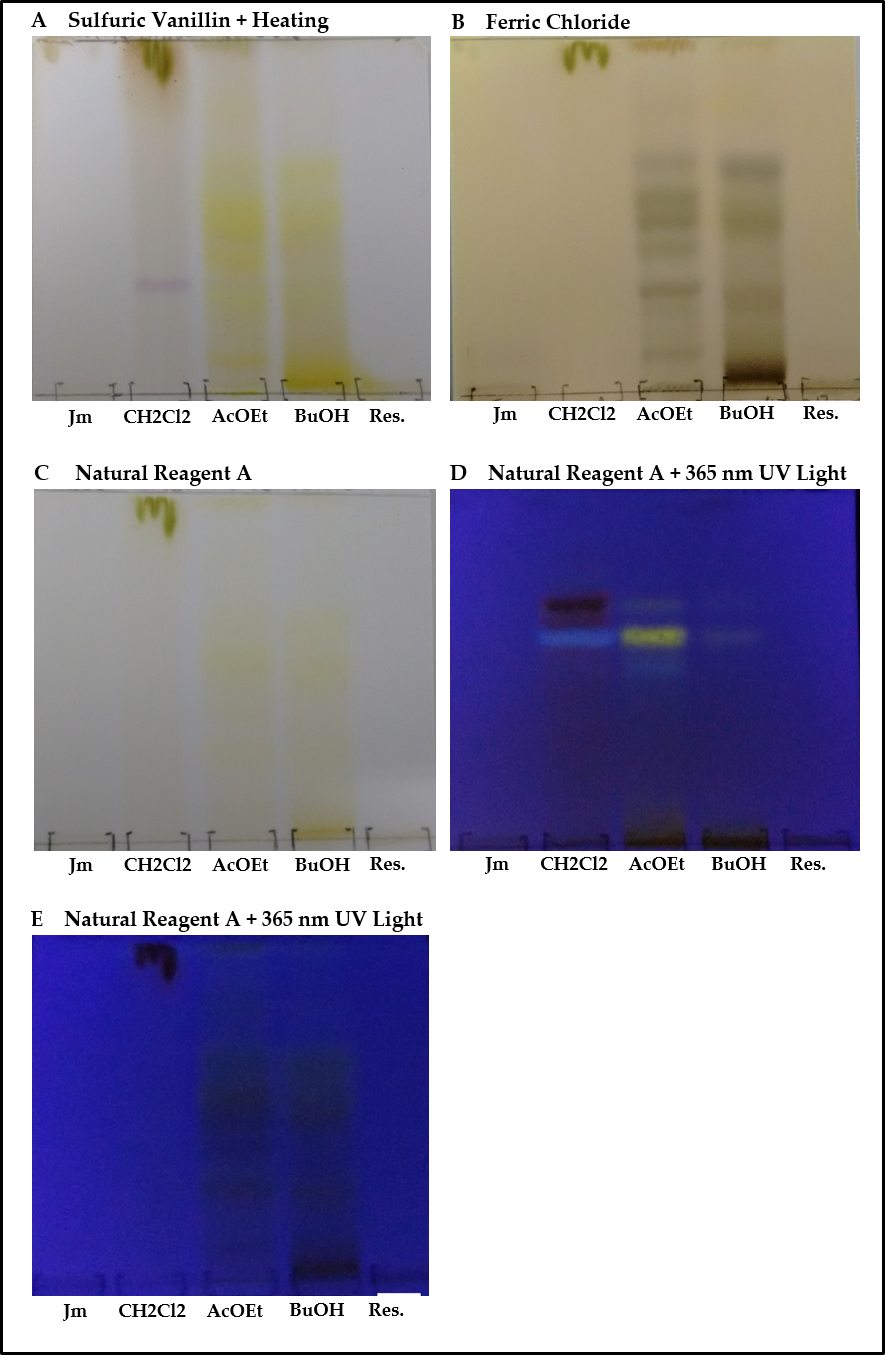


Figure S2: Thin Layer Chromatography. Photography by Jacyra A.S. Gomes.


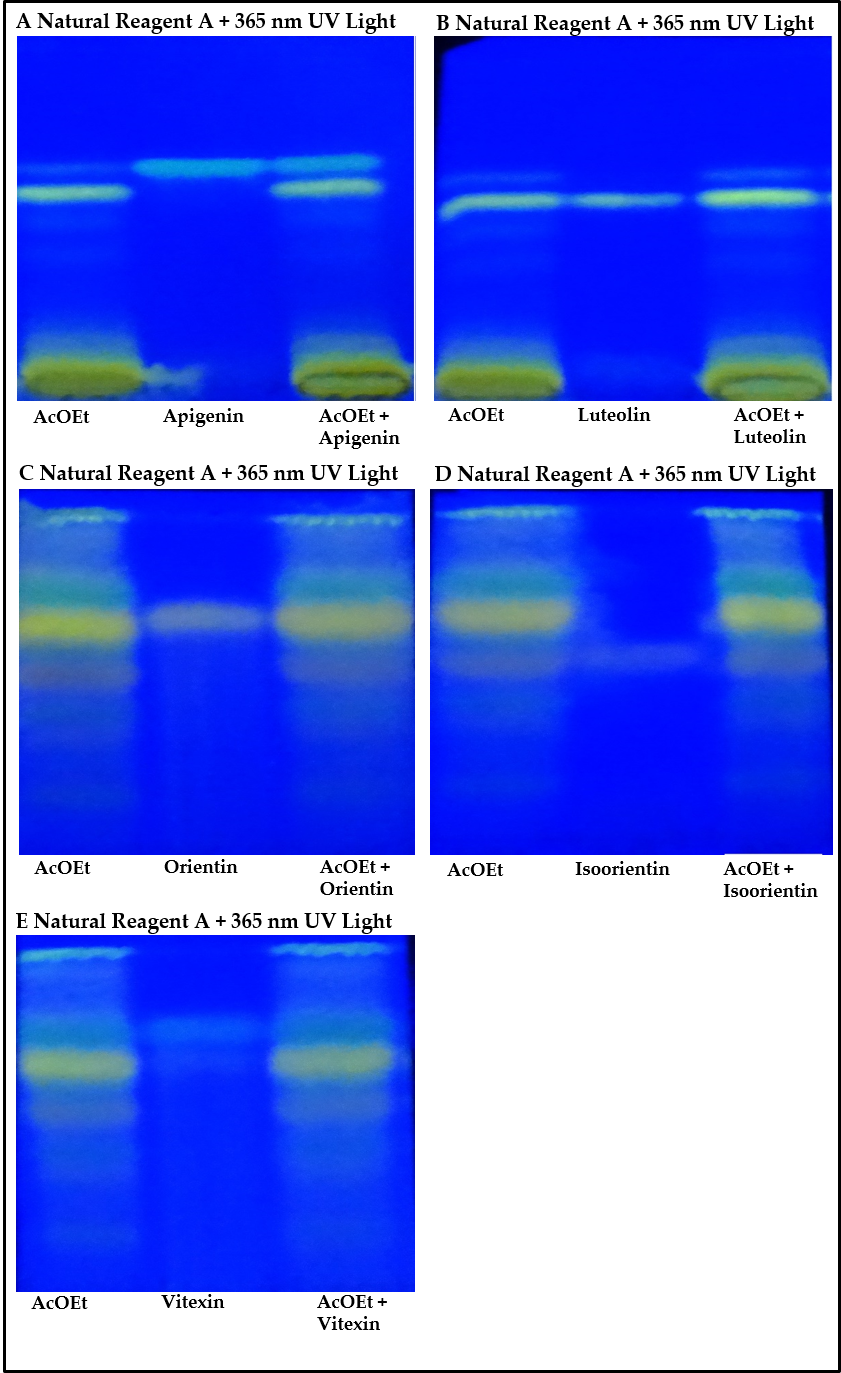


Figure S3: Co-Thin Layer Chromatography. Photography by Jacyra A.S. Gomes.
